# Mutations in the interleukin receptor *IL11RA* cause autosomal recessive Crouzon-like craniosynostosis

**DOI:** 10.1002/mgg3.28

**Published:** 2013-08-19

**Authors:** Katharina Keupp, Yun Li, Ibrahim Vargel, Alexander Hoischen, Rebecca Richardson, Kornelia Neveling, Yasemin Alanay, Elif Uz, Nursel Elcioğlu, Martin Rachwalski, Soner Kamaci, Gökhan Tunçbilek, Burcu Akin, Joachim Grötzinger, Ersoy Konas, Emin Mavili, Gerhard Müller-Newen, Hartmut Collmann, Tony Roscioli, Michael F Buckley, Gökhan Yigit, Christian Gilissen, Wolfram Kress, Joris Veltman, Matthias Hammerschmidt, Nurten A Akarsu, Bernd Wollnik

**Affiliations:** 1 Center for Molecular Medicine Cologne (CMMC), University of Cologne50931, Cologne, Germany; 2 Institute of Human Genetics, University of Cologne50931, Cologne, Germany; 3 Cologne Excellence Cluster on Cellular Stress Responses in Aging-Associated Diseases (CECAD), University of Cologne50931, Cologne, Germany; 4 Department of Plastic and Reconstructive Surgery, Hacettepe University Medical Faculty06100, Ankara, Turkey; 5 Department of Plastic and Reconstructive Surgery, Medical Faculty, Kirikkale University71100, Kirikkale, Turkey; 6 Department of Human Genetics, Radboud University Nijmegen Medical Centre6500HB, Nijmegen, The Netherlands; 7 Department of Physiology and Pharmacology, University of BristolBS8 1TD Bristol, U.K; 8 Department of Pediatrics, Pediatric Genetics Unit, Hacettepe University Medical Faculty06100, Ankara, Turkey; 9 Department of Pediatrics, Pediatric Genetics Unit, Acibadem University34457, İstanbul, Turkey; 10 Department of Medical Genetics, Gene Mapping Laboratory, Hacettepe University Medical Faculty06100, Ankara, Turkey; 11 Department of Biology, Duzce University81620, Duzce, Turkey; 12 Department of Pediatric Genetics, Marmara University Medical Faculty34668, Istanbul, Turkey; 13 Department of Orthodontics, Hacettepe University Faculty of Dentistry06100, Ankara, Turkey; 14 Medical Faculty, Institute of Biochemistry, University of Kiel24118, Kiel, Germany; 15 Medical Faculty, Institute of Biochemistry and Molecular Biology, RWTH Aachen University52074, Aachen, Germany; 16 Department for Neurosurgery, Medical Faculty, University of Würzburg97070, Würzburg, Germany; 17 Department of Haematology and Genetics, South Eastern Area Laboratory Services2031, Sydney, Australia; 18 Medical Faculty, Institute of Human Genetics, University of Würzburg97047, Würzburg, Germany; 19 Institute of Developmental Biology, University of Cologne50674, Cologne, Germany

**Keywords:** Autosomal recessive craniosynostosis, Crouzon, *FGFR2*, *IL11RA*, tooth erruption, supernumerary teeth

## Abstract

We have characterized a novel autosomal recessive Crouzon-like craniosynostosis syndrome in a 12-affected member family from Antakya, Turkey, the presenting features of which include: multiple suture synostosis, midface hypoplasia, variable degree of exophthalmos, relative prognathism, a beaked nose, and conductive hearing loss. Homozygosity mapping followed by targeted next-generation sequencing identified a c.479+6T>G mutation in the interleukin 11 receptor alpha gene (*IL11RA*) on chromosome 9p21. This donor splice-site mutation leads to a high percentage of aberrant *IL11RA* mRNA transcripts in an affected individual and altered mRNA splicing determined by in vitro exon trapping. An extended *IL11RA* mutation screen was performed in a cohort of 79 patients with an initial clinical diagnosis of Crouzon syndrome, pansynostosis, or unclassified syndromic craniosynostosis. We identified mutations segregating with the disease in five families: a German patient of Turkish origin and a Turkish family with three affected sibs all of whom were homozygous for the previously identified *IL11RA* c.479+6T>G mutation; a family with pansynostosis with compound heterozygous missense mutations, p.Pro200Thr and p.Arg237Pro; and two further Turkish families with Crouzon-like syndrome carrying the homozygous nonsense mutations p.Tyr232* and p.Arg292*. Using transient coexpression in HEK293T and COS7 cells, we demonstrated dramatically reduced IL11-mediated STAT3 phosphorylation for all mutations. Immunofluorescence analysis of mouse Il11ra demonstrated specific protein expression in cranial mesenchyme which was localized around the coronal suture tips and in the lambdoidal suture. In situ hybridization analysis of adult zebrafish also detected *zfil11ra* expression in the coronal suture between the overlapping frontal and parietal plates. This study demonstrates that mutations in the *IL11RA* gene cause an autosomal recessive Crouzon-like craniosynostosis.

## Introduction

The development of the craniofacial skeleton and calvarial sutures is a fundamental and complex biological process, whose cellular and molecular mechanisms are incompletely understood. Sutures are key players in the regulation of the growth and morphogenesis of early skull and brain development. Six cranial sutures are present in the human cranium: two coronal, one sagittal, two lambdoid, and one metopic (Cohen and MacLean 2000). Alterations in cellular interactions and signaling between the sutures may lead to craniosynostosis, a premature fusion of one or more of the cranial sutures during skull development. Early suture fusions (single or multiple) lead to distortions of skull development in the direction of the open sutures and are frequently associated with increased intracranial pressure, impaired blood flow, as well as hearing and vision impairments. Moreover, the pressure on the growing cerebral cortex results in an increased risk of intellectual disability (Renier et al. 1982).

Craniosynostosis is one of the most common craniofacial malformations being present in approximately 1 in 2000 live births (Cohen 1979; Lajeunie et al. 1995) and occurs either in rare syndromic, or more frequently, in nonsyndromic forms with isolated synostosis. Syndromic forms of craniosynostosis are frequently associated with limb malformations, but phenotypic associations can include a broad spectrum of features with more than 180 different syndromes associated with craniosynostosis. Although the majority of cases are sporadic, approximately 10% show familial recurrence (Cohen 2002). Monogenic forms of craniosynostosis are usually inherited in an autosomal dominant manner with highly penetrant mutations in *FGFR1-3* and *TWIST1* causing the most recognizable syndromes including Apert (MIM 101200), Crouzon (MIM 123500), Pfeiffer (MIM 101600), Antley-Bixler (MIM 207410), Muenke (MIM 602849), and Seathre-Chotzen (MIM 101400) syndromes. Crouzon syndrome (CS) is characterized by frequent bicoronal synostosis and occasional pansynostosis, hypertelorism, exophthalmos, divergent strabismus, a beaked nose, short philtrum, hypoplastic maxilla, and relative prognathism. Malformations of the extremities are more subtle in patients with CS than in Pfeiffer and Apert syndromes and thus show clinical utility in distinguishing CS from other craniofacial syndromes with overlapping cranial phenotypes (Kaler et al. 1982; Murdoch-Kinch and Ward 1997; Mooney and Siegel 2002). Variable inter- and intrafamilial expressivity of CS is well documented. Typically CS is inherited in an autosomal dominant fashion due to heterozygous activating mutations in *FGFR2* (MIM 176943). Although autosomal recessive inheritance of CS has previously been reported this has received limited attention due to the rarity of large families with this mode of inheritance (Cross and Opitz 1969; Juberg and Chambers 1973).

Here, we report consanguineous families with a Crouzon-like phenotype presenting with multiple suture synostosis, exophthalmos, midfacial hypoplasia, and prognathism without limb malformations. Clinical findings are indistinguishable from autosomal dominant CS, although intra- and interfamilial variation does exist. Homozygosity mapping and targeted next-generation sequencing identified missense and nonsense mutations in the *IL11RA* gene on chromosome 9p21.1-p13.2 impairing STAT3-related downstream signaling. Moreover, we demonstrate that mutations in *IL11RA* also underlie early suture closures in pansynostosis. Our data provide exciting evidence for the involvement of interleukin 11 signaling in cranial suture development and disease.

## Materials and Methods

### Clinical studies

**Figure 1:**
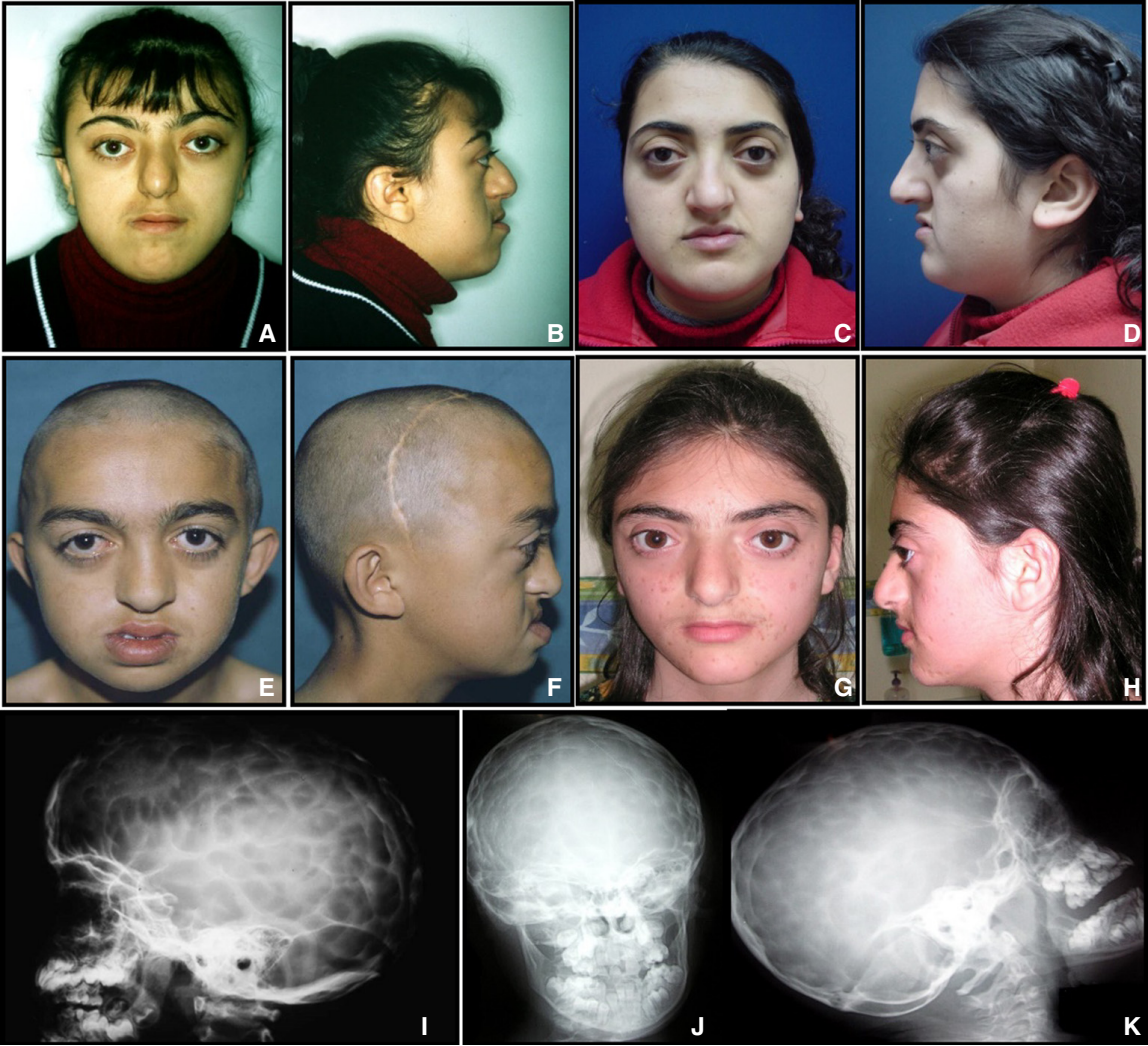
Craniosynostosis phenotypes linked to *IL11RA* mutations. (A-H) Facial views of representative cases of the Turkish CRS1 family with Crouzon-like craniosynostosis. (A and B) Subject IV:5. Facial appearance at 17 years. (C and D) Subject IV:11 at 16 years old. (E and F) Affected individual of the CRS3 family carrying the p.Arg292* mutation at approximately 9 years of age. All three subjects were treated by craniotomy at an early age. (G and H) Affected individual of the CRS4 family with p.Tyr232* mutation at 12 years of age. (I-K) Skull X-ray with increased digital markings and maxillary hypoplasia present in CRS3.

**Figure 2:**
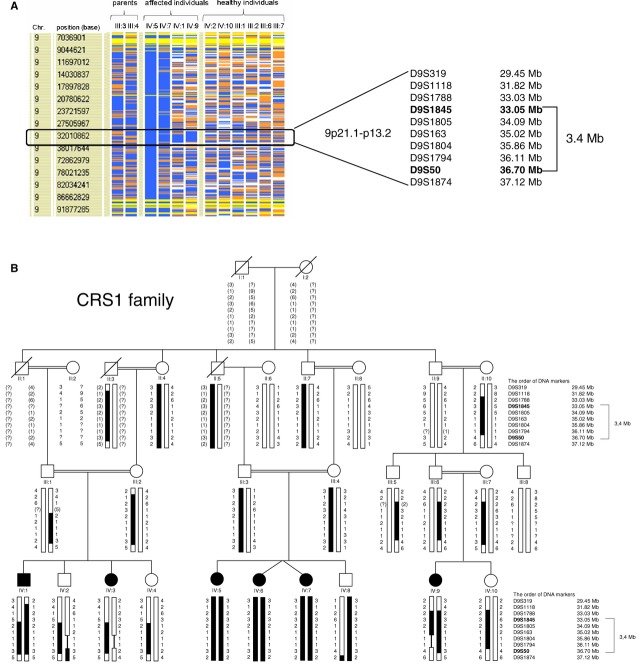
Mapping data of CRS1 family manifesting autosomal recessive Crouzon-like craniosynostosis. (A) Schematic representation of homozygosity data of the chromosome 9p21-p12 region. Homozygous genotypes identical to the genotype data obtained from index case IV:5 (also see pedigree in (B)) are shown in blue. Contrasting homozygous genotypes are shown in white whereas heterozygous SNPs appear orange. Noninformativeness as a result of heterozygous genotypes in parent–child trios is indicated in yellow. A single homozygous segment of approximately 11 Mb in size between position 27–38 Mb was observed and is marked in the rectangle. The genotyping results of microsatellite DNA markers shown on the right are presented in the pedigree below. (B) Pedigree and haplotype analysis of the autosomal recessive CS family. Genotyping data and haplotype bars for chromosome 9p markers are shown below the symbol for each individual. Black bars denote the disease-associated region. Genotypes in the bracket are the most likely genotypes that are deduced from their children. Thin bars represent noninformative genotypes. The critical recombination events positioned the disease allele between DNA markers *D9S1845* and *D9S50* (marked as bold) within a 3.4 Mb critical interval on chromosome 9p21-p12.

Three siblings from the index family (Figs. 1A–D and 2B, individuals IV:5, IV:6, IV:7) were identified from the Hacettepe University Craniomaxillofacial Study Group registry. All three affected individuals originated from Antakya, Hatay, Turkey, a region with an increased rate of consanguinity. A field study was conducted by NAA, IV, and SK to evaluate relatives and pedigree construction. The complete pedigree structure contained over 427 individuals and various malformations such as X-linked nystagmus (Kaplan et al. 2008), autosomal dominant hypodontia, autosomal recessive Carnevale syndrome, and craniosynostosis. Only the craniosynostosis cases and their families were included in this study. Cases with Crouzon-like syndrome were scattered over the various branches of this isolate. Affected members, their parents, and surviving grandparents were examined (Figs. 1 and 2). Blood samples were collected and DNA was extracted following standard protocols after informed consent was received. Institutional ethical board approvals for the research project were obtained.

### Molecular studies

DNA samples from four affected individuals, their parents, and unaffected siblings were genotyped using single nucleotide polymorphisms (SNPs) with the GeneChip Mapping 10K Array Set (Affymetrix, Santa Clara, CA). Genomic DNA (250 ng) was digested by XbaI, followed by adaptor ligation and PCR amplification with primers provided by the manufacturer (Affymetrix). PCR amplification products were then purified using the Qiagen MinElute 96 protocol (Qiagen Inc, Valencia, CA), fragmented by DNase I, labeled with terminal deoxynucleotidyltransferase and hybridized to the Mapping 10K Xba Chips (Affymetrix). For homozygosity mapping, genotype files (*CHP files*) were generated by the Affymetrix GTYPE software and transferred to the VIGENOS (Visual Genome Studio) program (Hemosoft Inc, Ankara, Turkey) which allows the analysis of genome-wide data in comprehensible visual screens (Kayserili et al. 2009). Haplotypes indicating homozygosity by descent were compared with those of the index patient IV-5 (Fig. 2A). For microsatellite genotyping, several polymorphic DNA markers were selected from the critical region on chromosome 9p21-p13. PCR amplification, followed by denaturing polyacrylamide gel electrophoresis (6%–7%) and silver staining methods were used to separate and visualize the alleles. Gels were manually photographed (APC film; Promega, Madison, WI) and genotyped. The MLINK component of the LINKAGE program (FASTLINK, version 3) was used to analyze the linkage data (Lathrop and Lalouel 1984; Lathrop et al. 1984; Cottingham et al. 1993; Schaffer et al. 1994) under the assumption of an autosomal recessive model with complete penetrance.

### Targeted next-generation sequencing

The entire 3.6 Mb region on chromosome 9 (chr9:33,146,801-36,792,821), between markers *D9S1845* and *D9S50,* was targeted by array-based sequence capture followed by next-generation sequencing. After stringent probe selection by NimbleGen (Roche NimbleGen, Madison, WI) with uniqueness testing by the Sequence Search and Alignment by Hashing Algorithm (SSAHA), a total of >2.4 Mb of nonrepeat masked sequences were represented on the array, representing 385,000 oligonucleotide probes targeting the regions of interest. Sequence capture was performed in accordance with the manufacturer's instructions (Roche NimbleGen), with the use of the Titanium optimized protocol as described previously (Nikopoulos et al. 2010; Vermeer et al. 2010). In brief, 5 μg of proband genomic DNA was used for library preparation prior to sequence-capture hybridization. A final amount of 3 μg prehybridization ligation-mediated PCR-amplified DNA was hybridized to the customized array, eluted after 72 h of hybridization and amplified by post hybridization LM-PCR. The amplified captured sample was then used as the input for emulsion PCR amplification and subsequent sequencing by the Roche 454 GS FLX sequencer with Titanium series reagents.

### Mutation screening

Sequencing of the *IL11RA*, *IL11*, *IL6*, and *GP130* genes was performed using ABI 310 or ABI 3100 Genetic Analyzers (Applied Biosystems, Foster City, CA) following standard protocols. All of the identified mutations were resequenced in independent experiments, tested for cosegregation with the phenotype within the families, and then screened in 100 healthy control individuals from Turkey by PCR or restriction digestion (BstXI for the c.710G>C mutation, RsaI for c.874 C>T, HpaI for c.696 C>A), direct sequencing (c.479+6T>G) or mutation-specific primer amplification (c.598C>A). A further 74 control individuals from the same village of the original CRS1 family near Antakya were screened for c.479+6T>G by direct sequencing analysis. Primer sequences and conditions are provided in Table S1.

### Patient cDNA and in vitro exon-trapping analyses

Patient RNA was extracted from fresh blood using the Paxgene Blood RNA system (Qiagen, Hilden, Germany). RT-PCR was performed via RevertAid First Strand cDNA synthesis Kit (Fermentas, St. Leon-Rot, Germany). RT-PCR products were amplified by standard PCR using *IL11RA* specific primers, visualized by agarose gel electrophoresis and used for direct sequencing. Primers for amplification and sequencing were designed according to the reference sequences for exons 2 and 7 of *IL11RA*. In vitro analysis of splice mutation c.479+6T>C was performed by the Exon-Trapping System (Gibco Invitrogen, Karlsruhe, Germany). Genomic fragments of the wildtype and the mutant variant of *IL11RA* including exon 4 and 5 and the flanking intronic sequences were cloned into the pSPL3 expression vector using the EcoRI and EcoRV restriction sites. HEK293T cells were transiently transfected with wild type and mutant constructs followed by RNA extraction using RNeasy Kit (Qiagen) and reverse transcription via RevertAid First Strand cDNA synthesis Kit (Fermentas).

### IL11RA expression constructs

The pSVL-sIL11Rflag vector described previously (Pflanz et al. 1999), was used as the template for the generation of IL11RA expression constructs. The human cDNA of the soluble isoform of *IL11RA* fused to a C-terminal Flag tag was amplified via PCR and cloned into the pRK5 expression vector using NotI and HindIII restriction sites. The identified mutations (p.Pro200Thr, p.Arg237Pro, p.Tyr232*, and p.Arg292*) were introduced by site-directed PCR mutagenesis with primers containing the specific nucleotide substitutions.

### Cell culture and transient transfection

HEK293T and COS7 cells were cultured in Dulbecco′s Modified Eagle Media (DMEM) supplemented with 10% fetal bovine serum (FBS; Gibco) and antibiotics. Cells were transiently transfected with vectors containing wild type and mutant variants of *IL11RA* cDNA or genomic fragments of *IL11RA* using Lipofectamine 2000 (Invitrogen) following the manufacturer's instructions.

### Western blot analysis

Eighteen hours after transfection, cells were activated by the addition of 0.5 μg IL11 (Peprotec, Rocky Hill, NJ) into 8 mL growth medium for 10 minutes at 37°C. After incubation, activated and nonactivated control cells were harvested and total proteins solubilized using ice-cold lysis buffer. The total protein concentration of extracts was determined using the BCA Protein Assay Kit (Pierce Protein Research Products; Thermo Fischer Scientific, Rockford, IL), and 15 μg of total protein from each sample was separated by 4–12% SDS-PAGE and blotted onto nitrocellulose membranes (GE Healthcare, München, Germany). Protein detection was performed using antibodies against pStat3 (Santa Cruz Biotechnology Inc, Santa Cruz, CA). Equal protein loading and transfection efficiency was confirmed by reprobing of the membranes with antibodies against Flag and β-Actin. Peroxidase conjugated secondary antibodies were purchased from Santa Cruz and blots were developed using an enhanced chemiluminescence system, ECL Plus (Amersham, UK), followed by exposure on autoradiographic film (GE Healthcare).

### Expression analysis

For histological analysis, P0 wild-type mouse heads were embedded in tissue freezing medium (Leica Biosystems, Nussloch, Germany) and frozen by immersion in isopentane precooled in liquid nitrogen. Twelve micrometer cryosections were stained with hematoxylin and eosin (Merck, Darmstadt, Germany). For immunofluorescence analysis, 12 μm cryosections were blocked in 1× PBS containing 0.5% TritonX and 10% Fetal Calf Serum and probed with an antibody raised against Il11ra (1:20, (N-20) sc-993, Santa Cruz). Antibody binding was visualized with a Cy3-conjugated secondary antibody (Invitrogen) and the sections counterstained with DAPI (Invitrogen).

### In situ hybridization

In situ hybridizations were performed using probes generated from cloned cDNA fragments of zebrafish il11ra. Probes were synthesized from linearized plasmids using the Roche digoxigenin RNA synthesis kit. Adult zebrafish (6–12 months old) were fixed in 4% paraformaldehyde, decalcified in 0.5 mol/L EDTA (pH 7.4) for 5 days, dehydrated, cleared, embedded in paraffin, and sectioned at 8 μm. In situ hybridizations were performed as previously described (Moorman et al. 2001) with the following modifications: prehybridization and hybridization were performed at 62°C and BMPurple (Roche, Mannheim, Germany) was used for chromogenic detection.

## Results

The CRANIRARE consortium (http://www.cranirare.eu) is an E-RARE funded network and represents an integrated clinical and molecular approach for craniofacial malformations aiming to identify the molecular pathogenesis for various craniofacial malformations. The strategy to identify novel genes within the presented study was based primarily upon a large collection of patients from the Hacettepe University Craniomaxillofacial Study Group registry, which principally includes syndromic forms of craniosynostosis. A prescreen for mutations in the craniosynostosis genes *FGFR1*, *FGFR2*, *FGFR3*, and *TWIST* was performed and mutations identified in approximately 50% of cases (B. Wollnik, pers. comm., 2010).

### An autosomal recessive Crouzon-like phenotype

**Figure 3:**
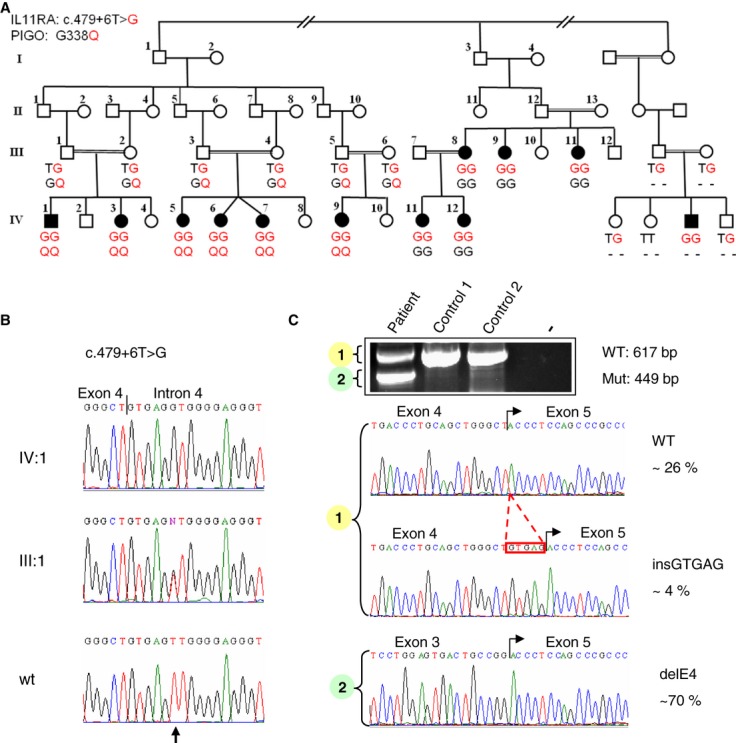
Identification of a donor splice-site mutation in *IL11RA*. (A) Pedigree of the large Turkish kindred with Crouzon-like craniosynostosis. Capital letters beneath each symbol describe the genotypes of family members for the two variations identified by targeted next-generation sequencing in *IL11RA* and *PIGO*. Amino acid and nucleotide substitutions are shown in red. (B) Sequence chromatograms showing the identified c.479+6T>G mutation in *IL11RA* in the affected individual IV:1, heterozygous carrier III:1, and a wild-type (wt) control. The arrow indicates the nucleotide substitution on the sixth position behind the exon intron boundary. (C) mRNA analysis of *IL11RA* transcripts in an affected individual carrying the c.479+6T>G mutation in the homozygous state. RNA was reverse transcribed and after PCR amplification separated on a 1% agarose gel resulting in two visible bands containing three fragments (patient lane). The upper band (∼600 bp) contained a WT fragment and an aberrant transcript which includes additional 5 bp generated by the use of an alternative donor site introduced by the mutation. The lower band (∼450 bp) represents an aberrant transcript generated by skipping of exon 4. Frequencies of transcripts are indicated at the right and represent the analysis of 21 clones after subcloning and sequencing of fragments.

The index family (Fig. 1) was identified due to CS-like findings segregating with an autosomal recessive mode of inheritance. Consistent findings were multiple suture synostosis, midfacial hypoplasia, variable exophthalmos, and relative prognathism (Table 1). A beaked nose and conductive hearing loss are features observed in some family members. The index case (Figs. 1A and B, 2B individual IV:5) is a 17-year-old female with complex suture synostosis, brachycephaly, and hearing loss with an initial diagnosis of CS. Her twin sisters (Fig. 2B, individuals IV:6 and IV:7) had similar craniofacial malformations while both parents were unaffected. The index case was treated with a strip craniectomy at the age of 3 years and her twin sisters had cranial surgery at the age one. A total of twelve individuals with Crouzon-like phenotype were identified in various branches of the pedigree (Fig. 3A and Table 1) following practitioner review. Two individuals (Fig. 3A, IV:11 and III:8) had dental extractions due to failure of tooth eruption.

**Table 1:** Clinical findings in patients carrying *IL11RA* mutations

Family	Cases	Mutation	Craniosynostosis	Midfacial hypoplasia	Exophthalmos	Hypertelorism	Hearing loss	Parrot-beaked nose	Occlusion	Crowded dentation	Other findings
CRS1	IV:1	c.479+6T>G	Yes	Yes	Yes	Yes	No	No	Class III	?	
	IV:3	c.479+6T>G	Yes	Yes	Mild	No	No	No	Class III	?	
	IV:5	c.479+6T>G	Yes	Yes	Yes	No	Yes	Yes	Class III	No	
	IV:6	c.479+6T>G	Yes	Yes	Yes	No	Yes	Yes	Class III	No	
	IV:7	c.479+6T>G	Yes	No	Yes	Yes	Yes	No	Class I	No	
	IV:9	c.479+6T>G	Yes	No	Mild	No	No	No	Class I	?	
	IV:11	c.479+6T>G	Yes	Yes	Yes	No	No	Yes	Class III	?	Infantile paralysis poliomyelitis
	IV:12	c.479+6T>G	Yes	Yes	Yes	No	No	Nasal reconstruction	Edge-to edge bite	Failure of teeth erruption	
	IV:17	c.479+6T>G	Yes	Yes	Yes	No	No	Yes	Class III	No	
	III:8	c.479+6T>G	Yes	Yes	Mild	No	Yes	No	Edge-to edge bite	Failure of teeth erruption	
	III:9	c.479+6T>G	Yes	Yes	Yes	Yes	No	No	Class III	No	
	III:11	c.479+6T>G	Yes	Yes	Yes	No	No	No	Class III	No	
CRS2	II:1 II:2	p.Pro200Thr/p.Arg237Pro p.Pro200Thr/p.Arg237Pro	Yes Yes	Yes Yes	Mild Mild	Yes Yes	No No	No No	Edge-to-edge bite	No No	Mild developmental delay
CRS3	II:2	p.Arg292^*^	Yes	Yes	Yes	No	No	No	Class III	?	
CRS4	II:1	p.Tyr232^*^	Yes	Yes	Yes	Yes	No	No	Edge-to edge bite	Failure of teeth erruption	Hypermobility, immune deficiency
CRS5	II:1	c.479+6T>G	Yes	No	No	No	No	No	Class I	No	

?: no examination record is available.

### Identification of a homozygous splice-site mutation in *IL11RA*

A total of 12 individuals from this family were genotyped using the Affymetrix GeneChip Human Mapping 10K SNP Array. Genotype files were generated with Affymetrix GTYPE software and transferred to the VIGENOS (Visual Genome Studio) program (Hemosoft) (Kayserili et al. 2009) to identify the minimum region of shared homozygosity (Fig. 2A). A single homozygous region on chromosome 9p21-p12 was found in all the affected individuals, for which the parents and healthy siblings were heterozygous. Subsequently, typed microsatellite markers for this chromosomal regions included additional family members (Fig. 2B). Homozygosity was confirmed and recombinations between markers *D9S1845* and *D9S50* defined a critical interval of 3.4 Mb containing 78 annotated genes. Linkage analysis obtained a maximum pairwise lod score of 2.306 at Theta = 0 cM between the disease allele and the DNA marker D9S1805 (data not shown). Subsequently, array-based sequence capture of the complete 3.4 Mb region was performed, followed by next-generation sequencing on a one-quarter plate of a Roche 454 sequencing run. Of the 61 million sequencing reads approximately 50% of the sequence data mapped back to, or near, the targeted region of chromosome 9p21-p12 and the average of coverage for the region was 11-fold. 98.4% of all targeted bases were sequenced at least once and >83% of all targeted bases were covered at least fivefold.

Among the identified homozygous variants (Table 2) only two were not annotated as SNPs and were located within either exonic sequences or splice sites: a nonsynonymous coding variant, p.Gly338Gln, in *PIGO* and the c.479+6T>G variant of the donor splice site of exon 4 of *IL11RA*. Both variants were confirmed by Sanger sequencing, but only the c.479+6T>G variant (Fig. 3B) cosegregated with the disease in the extended pedigree and was present in all 12 affected individuals in various branches of the family (Fig. 3A). The c.479+6T>G mutation was not present in 100 Turkish controls. Further evidence that this splice-site mutation is likely to be the pathogenic allele came from the finding that this mutation leads to a high percentage of aberrant *IL11RA* mRNA transcripts in an affected individual (Fig. 3C) and alters normal splicing in an in vitro exon-trapping experiment (Fig. S1). It is noteworthy that the donor splice site of exon 4 is not completely abolished and approximately 25% of the normal transcript was observed in mRNA derived from one affected individual.

**Table 2:** Next generation sequencing variant statistics

Filter applied	Number of variants
All variants called	2529
Mapping to target region on chr.9	1958
Not previously known SNP (dbSNP129)	105
Homozygous variant (>80% variant reads)	74
Coding/splice site variant	2

In order to determine the carrier frequency of the *IL11RA* c.479+6T>G mutation, we tested a total of 74 individuals from the same village. From these data, we included parents and spouses only (30 individuals) to calculate the mutant allele frequency. Expected carrier frequency (2pq) was approximately 0.30 for this village (normal T-allele frequency (p) = 0.816 and mutant G-allele frequency (q) = 0.184). Following identification of the *IL11RA* c.479+6T>G mutation, we confirmed the presence of this allele in an additional consanguineous family with three affected siblings with Crouzon-like findings from a nearby village of Antakya. Although no clear relationship between this and the index family could be established, the identical c.479+6T>G mutation was identified by direct sequencing. In conclusion a total of 15 affected individuals with the *IL11RA* c.479+6G>T mutation suggests a founder effect in the Antakya region of Turkey.

### Additional *IL11RA* mutations in Crouzon-like syndrome and pansynostosis

**Figure 4:**
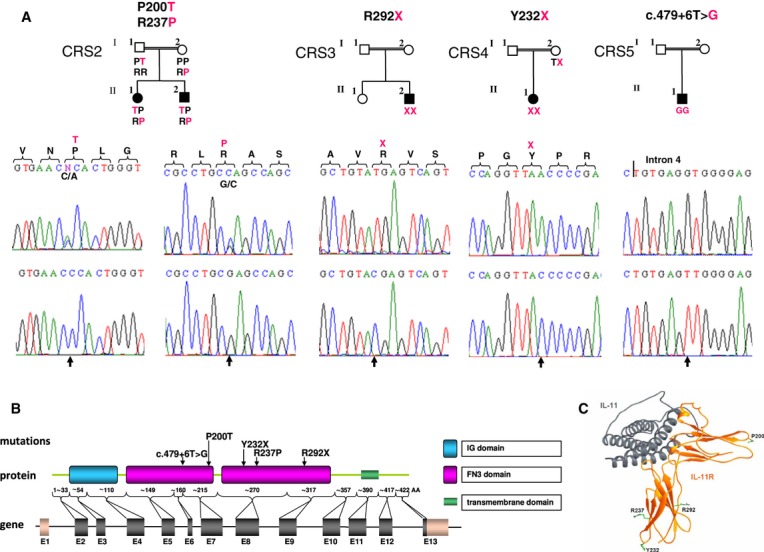
Additional *IL11RA* mutations. (A) Pedigrees and sequence chromatograms of additional *IL11RA* mutations. Capital letters above the pedigrees describe the amino acid changes found in affected family members. Substitutions are marked in red. (B) Schematic representation of the genetic and molecular structure of IL11RA and positions of identified mutations within IL11RA domains. (C) Predicted protein structure of the IL11-IL11RA receptor complex. Amino acids at the positions of the identified missense (Pro200, Arg237) and nonsense mutations (Tyr232, Arg292) are shown.

All 13 coding exons of the *IL11RA* gene were screened by DNA sequencing in a cohort of 79 patients with a diagnosis of either CS, pansynostosis, or nonclassified syndromic craniosynostosis. The *IL11RA* c.479+6T>G mutation was also identified in a consanguineous patient from Germany of Turkish origin. Interestingly, this patient presented with pansynostosis suggesting that mutations in *IL11RA* may display clinical variability (Table 1). Pansynostosis was also the leading diagnosis in two affected siblings from a nonconsanguineous family also from Turkey (Table 1), both of whom had the following compound heterozygous missense mutations (Fig. 4A): *IL11RA* c.598C>A (p.Pro200Thr) paternal and c.710G>C (p.Arg237Pro) maternal. Both mutations are located within the FN3 domain of the IL11RA protein (Fig. 4B,C). The homozygous nonsense mutations, c.696C>A (p.Tyr232*) and c.874C>T (p.Arg292*), were identified in two additional Turkish families with Crouzon-like syndrome (Table 1 and Fig. 4A). These mutations are predicted to truncate the IL11RA protein within the FN3 domain, leading to a loss of the C-terminal transmembrane domain (Fig. 4B,C). Neither of these mutations were found in 100 healthy Turkish control individuals.

### Impaired IL11RA signaling caused by identified mutations

**Figure 5:**
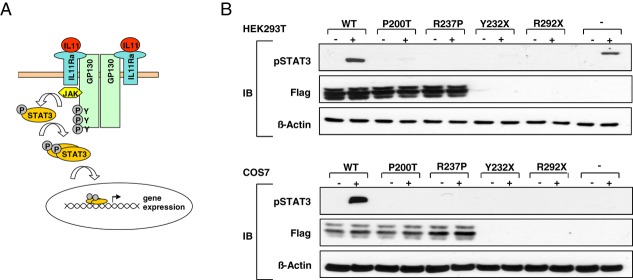
Impaired IL11RA signaling causes craniosynostosis. (A) Simplified schematic view of the signaling pathway involving IL11RA. Binding of IL11 to IL11RA is thought to trigger homodimerization of the GP130 receptor and transphosphorylation of tyrosines of GP130 through the associated JAKs. Subsequently, STAT3 is activated by tyrosine phosphorylation. (B) Western blot analysis of IL11-induced phosphorylation of STAT3 in transiently transfected HEK293T and COS7 cells. Cells were activated with IL11 (+) or left unactivated (-). Transfection efficiency and equal protein amounts were confirmed by reprobing of the membrane with antibodies against Flag and ß-Actin. C-terminal fused Flag-tag was neither detectable in proteins with premature stop signals nor in untransfected control cells.

IL11RA is a known receptor involved in interleukin signaling. After the binding of its ligand, IL11, the receptor binds to the cytokine signal transducer GP130, leading to dimerization and subsequent transphosphorylation of intracellular tyrosines within the cytoplasmic domain through the associated Janus kinases (Jaks) (Dahmen et al. 1998). One of the known downstream targets is STAT3, which is activated by phosphorylation in the signaling cascade (Fig. 5A) (MIM 102582). Using transient expression experiments in HEK293T and COS7 cells, we demonstrated that both the missense mutations and nonsense mutations of IL11RA reduced in vitro IL11-dependent STAT3 phosphorylation to undetectable levels (Fig. 5B). These results clearly implicate impaired interleukin 11 signaling as a novel pathogenic mechanism underlying Crouzon-like syndrome and pansynostosis.

### Mutation screen in IL11RA pathway genes

In order to test the hypothesis that mutations in additional genes encoding proteins involved in IL11RA signaling could also be associated with craniosynostosis, the five coding exons of *IL11* were sequenced in 46 patients with various forms of craniosynostosis as well as the five coding exons of *IL6* in 28 patients and the 14 coding exons of the *GP130* in 44 patients. No candidate causative mutations were identified, from which we conclude that alterations of these genes are not a frequent cause of syndromic craniosynostosis.

### Conserved expression of *Il11ra* in mouse and zebrafish sutures

**Figure 6:**
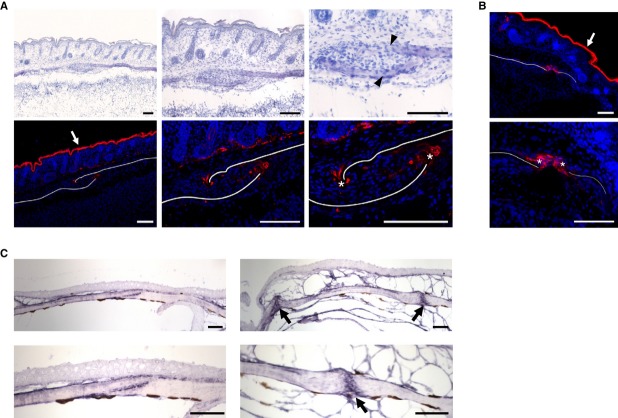
*Il11ra* expression in mouse and zebrafish sutures. (A-B) Sagittal cryosections of P0 mouse heads; scale bars: 100 μm. (A) Upper row, histological staining with hematoxylin and eosin shows the coronal suture (arrow heads) and surrounding tissue. Lower row, immunofluorescence staining displays Il11ra localization (red) in the granular layer of the skin (arrow) and around the coronal suture tips (asterisks). Nuclei were stained with DAPI (blue). White lines mark cranial bone plates. (B) Immunofluorescence staining of Il11ra (red) shows protein in the lambdoidal suture (asterisks). Nuclei were stained with DAPI (blue). White lines mark cranial bone plates. (C) In situ hybridization of *il11ra* mRNA on sagittal paraffin sections of adult zebrafish heads. *il11ra* is expressed within the coronal sutures (left) and anteriorly in craniofacial bone fusions (right, arrows) within areas of bone contact; scale bars: 100 μm.

Immunofluorescence analysis of mouse Il11ra demonstrated specific protein localization in cranial mesenchyme and in the granular layer of the epidermis of newborn mice (Fig. 6A,B). Histological analysis of P0 mouse cranial tissue revealed the normal structure of the coronal suture, formed by the overlapping frontal and parietal bones (Fig. 6A, upper panel). Immunofluorescence analysis of the murine ortholog of IL11RA demonstrated protein localization around the coronal suture tips (Fig. 6A, lower panel). Significant IL11ra protein levels could also be detected in the lambdoid suture in the posterior skull (Fig. 6B). Here, the suture is not formed by overlapping plates but by direct contact of the two opposing bones. Il11ra protein could be detected in the zone of bone contact between the plates forming the lambdoid suture (Fig. 6B). In situ hybridization analysis of adult zebrafish also confirmed cranial expression of the zebrafish ortholog of IL11RA (Fig. 6C). The zebrafish *il11ra* RNA was localized in the coronal suture, between the overlapping frontal and parietal plates (Fig. 6C, left panel). The arrangement of the skull plates in fish is very similar to that observed in mouse (Quarto and Longaker 2005). In the anterior part of the zebrafish head, two further joint-like structures of flat bones were detectable and specific *il11ra* expression was also detected in the areas of these bone contacts (Fig. 6C, right panel). The observed similarities between mouse and zebrafish cranial structure provides evidence for a conserved mechanism of suture formation in vertebrates. Furthermore, we can conclude a conserved involvement of IL11RA in suture development in both zebrafish and mouse.

## Discussion

Here, we demonstrate that mutations in the *IL11RA* gene cause autosomal recessive Crouzon-like craniosynostosis or syndromic pansynostosis with mild craniofacial phenotype via impaired interleukin-11 mediated signaling, based on mutations in five families.

Consistent findings in all affected family members of the CRS1 family were multiple suture synostosis, midfacial hypoplasia, variable exophthalmos, prognathism, a variably beaked nose, and conductive hearing loss. The craniosynostosis observed was complex and affected most sutures (pansynostosis) in these individuals with an initial diagnosis of CS. CS is a well-known entity and has been associated with heterozygous gain-of-function mutations in the FGFR2 receptor (MIM 176943). It is mainly characterized by frequent bicoronal synostosis with occasional pansynostosis, hypertelorism, exophthalmos, divergent strabismus, beaked nose, short philtrum, hypoplastic maxilla, and prognathism. This study confirms previous reports (Cross and Opitz 1969; Juberg and Chambers 1973) that an autosomal recessive form of a Crouzon-like craniosynostosis exists and shows that this can be caused by mutations in *IL11RA*. After initial gene identification, we screened all syndromic and nonsyndromic craniosynostosis cases in the CRANIRARE database and detected mutations only in cases classified as having CS. Therefore, we suggest that patients with Crouzon-like features and parental consanguinity should be first tested for *IL11RA* mutations. No additional skeletal anomalies affecting the cervical spine, wrist and limbs as well as no internal organ involvement was observed. We did not observe specific phenotypic differences between *IL11RA* and *FGFR2* caused craniosynostosis.

Nieminen et al. (2011) published a study showing that recessive mutations in *IL11RA* cause craniosynostosis, delayed tooth eruption, and supernumerary teeth. The authors state that one of the children in Family 2 had a Crouzon-like facial appearance which was not considered typical of any of the previously described craniosynostosis syndromes. In accordance with the clinical data presented by Nieminen et al., we observed class I and class II malocclusion and failure of tooth eruption in a large number of patients (Table 1). These findings are also consistent with IL11RA having a role in dental development.

It is known that heterozygous activating mutations in *FGFR2* can cause different phenotypes, such as Crouzon, Apert, Pfeiffer, or Antely-Bixler syndromes. The factors modifying the phenotypic outcome of *FGFR2* mutations are not well understood. Interestingly, we provide evidence that recessive mutations in *IL11RA* also show variable clinical expression (Table 1). As an example, both affected individuals of the CRS2 family presented with only pansynostosis, that is, without midfacial hypoplasia, exophthalmos, or relative mandibular prognathism, indicating that the clinical spectrum of *IL11RA* mutations ranges from pansynostosis to Crouzon-like craniosynostosis. The pansynostosis in this family is caused by compound heterozygous missense mutations, p.Pro200Thr and p.Arg237Pro, in IL11RA. Functional analysis of mutant proteins in two different cell systems clearly indicates that both mutations cause an impairment of IL11RA-mediated signaling as evidenced by the lack of downstream STAT3 activation (Fig. 5B). No functional differences in STAT3 activation were observed between the missense and nonsense mutations analyzed. Therefore, it is unlikely that variable phenotypes observed in our patients can be attributed to the different genotypes observed. This is further supported by the fact that the affected individual of the CRS5 family, who carries the homozygous c.479+6T>G mutation, which is also present in 15 Crouzon-like craniosynostosis cases from the region of Antakya, does present with Crouzon-like syndrome but has pansynostosis. It will be an important aim for future studies to identify molecular modifiers responsible for phenotypic variability.

Recent advances in massive parallel sequencing technologies have accelerated gene identification studies (Ng et al. 2010; Kalay et al. 2011). Here, targeted NGS of the complete linked critical region was used to sequence all 78 annotated genes within this region. Interestingly, only two variations of a putatively causative nature were detected (Table 2) of which only one cosegregated with the phenotype in the extended pedigree. These data nicely underscore the power of NGS applications, especially when mapping information in a family is available. We identified one homozygous splice-site mutation (c.479+6T>G), two homozygous nonsense mutations (p.Tyr232* and p.Arg292*), and compound heterozygosity for two missense mutations (p.Pro200Thr and p.Arg237Pro) in our patient cohort. All mutations identified in our study were shown to cause impairment of IL11RA function upon activation by IL11. We demonstrated that the c.479+6T>G donor splice-site mutation causes incomplete aberrant mRNA splicing, and that both missense and nonsense mutations fail to lead to STAT3 phosphorylation in response to IL11. Therefore, we conclude that impaired IL11-IL11RA signaling is the pathogenic mechanism underlying autosomal recessive Crouzon-like craniosynostosis and autosomal recessive pansynostosis. No mutations were identified in the *IL11, IL6,* and *GP130* genes in the CRANIRARE cohort.

Interleukin-11 (IL11) belongs to the interleukin-6-family of cytokines (Paul et al. 1990; Yin et al. 1992) and has mesenchymal cell specific activity, which includes bone marrow stromal cells and osteoblasts (Girasole et al. 1994). IL11 has a wide spectrum of biological functions and is involved in hematopoiesis, immune responses, neuronal processes, and bone metabolism (Musashi et al. 1991; Yin et al. 1992; Mehler et al. 1993; Du and Williams 1994, 1997). Il11ra plays a role in bone formation and remodeling and is expressed in both osteoblasts and mature osteoclasts. Moreover, bone-forming and bone-resorbing cells are potential targets of Il11 (Romas et al. 1996). During bone developmental processes, cytokines, including IL11, which binds to gpl30 receptor, can modulate stromal cell commitment to osteoblast differentiation pathways (Gimble et al. 1994). IL6 induced osteoblast maturation is mediated primarily by activation of the JAK/STAT signaling pathway (Bellido et al. 1997). IL11 also stimulates RANKL expression in osteoblasts and stromal cells, thereby indirectly promoting RANK-mediated osteoclastogenesis (Yasuda et al. 1998). Furthermore, IL11 signaling has direct effects on osteoclast precursors and stimulates osteoclast formation in a RANKL-independent mechanism (Kudo et al. 2003; Sims et al. 2005). Both direct and indirect IL11-stimulated osteoclast formation is a gp130-dependent process. However, RANKL-induced indirect osteoclast promotion occurs via a STAT3-mediated process (O'Brien et al. 1999), whereas direct effects on osteoclast formation are mediated by SHP2/Ras/MAPK signaling (Sims et al. 2004, 2005). Mice deficient for Il11ra (*Il11rα1*^*−*^*/*^*−*^) exhibit abnormal bone turnover and long bone structure and moreover, a reduction of osteoclast differentiation has been shown to be responsible for increased trabecular bone volume (Sims et al. 2005). Recently, disturbed cranial growth and suture activity in *Il11ra* knockout mice have also been described (Nieminen et al. 2011). In this study, expression analysis in newborn mice indicated a highly specific localization of Il11ra within the mesenchyme of the coronal suture. Interestingly, during suture formation in adult zebrafish, *il11ra* transcripts were also detectable in the intervening mesenchymal tissue, which provides further evidence for a highly conserved role of IL11RA during suture development in vertebrates.

The cranial skull vault anatomy and development is very similar in mouse and fish. For both mouse and zebrafish it is described that sutures – except the posterior frontal suture in mice – remain patent for the life of the animal (Opperman 2000; Quarto and Longaker 2005). The highly specific detection of Il11ra restricted to both ends of the cranial plates within the suture matrix in mice and expression of *il11ra* in a fibrous stripe of cellular material within the zebrafish coronal sutures reveal an involvement of Il11ra in separating the frontal and the parietal bones, which subsequently could prevent a fusion of these bones. This suggestion is supported by these genetic and clinical findings showing that mutations in *IL11RA* cause premature fusion of cranial sutures in patients with Crouzon-like syndromes and pansynostosis.

In our mouse expression study, Il11ra localized to a specific cell cluster within the mesenchyme, possibly representing a functionally distinct cell population. To date, it is not clear if alterations in Il11ra cause a dysfunction of osteoblasts or osteoclasts or an early developmental effect on mesenchymal precursor cells during osteoblast/osteoclast differentiation. Hyper-ossification defects as evident in the fused sutures of the patients are most likely due to increased osteoblast or decreased osteoclast activity. However, direct STAT3-mediated IL signaling, which according to our in vitro data is compromised by the IL11RA mutations, has been shown to have a stimulatory effect on osteoblasts, whereas direct stimulatory IL signaling on osteoclasts is mediated by other transduction pathways (see above). Therefore, we hypothesize that the pathogenesis of the disorder in IL11RA patients is caused by a disruption of the osteoblast-mediated, RANKL-dependent, indirect positive effect on osteoclastogenesis or osteoclast activity. However, other effects on osteoblasts independently of their regulatory role on osteoclasts also cannot be ruled out, such as an attenuated recruitment of osteoblasts from mesenchymal precursors during earlier development and a corresponding aberrantly high number of osteoblasts at the relevant stages.

In conclusion, our results provide evidence for a crucial and conserved role of IL11RA during craniofacial development and suture formation. We propose an inhibitory effect of Il11ra within sutures, thereby preventing their premature fusion, something which was also recently suggested in *Il11ra1*^*−*^*/*^*−*^ mice. In this study, it was shown that IL11 signaling has a negative regulatory effect on suture closure (Nieminen et al. 2011). Taken together, our data demonstrate a vital role for IL11-IL11RA signaling during craniofacial morphogenesis in humans, mouse, and zebrafish.

## Web Resources

UCSC Genome Bioinformatics, http://www.genome.ucsc.edu/

OMIM and Unigene, http://www.ncbi.nlm.nih.gov/

Ensembl, http://www.ensembl.org/index.html

CRANIRARE, http://www.cranirare.eu/

## Supporting Information

**Fig. S1.** In vitro exon-trapping analysis in HEK293T cells. (A-B) Transiently expressed splice vector pSPL3, which contained a genomic fragment of *IL11RA* including exon 4 and 5 and surrounding intronic sequences, resulted in two alternative splice products. Product 1 (upper band) contained a fragment with correctly spliced exon 4 and 5 of *IL11RA*, and was only present in cells transfected with WT constructs. In product 2 (lower bands) exon 4 is skipped and was present in cells expressing WT and mutant constructs containing splice mutation c.479+6T>G.

**Table S1.** Primer sequences used for amplification and sequencing of the *IL11RA* gene from genomic DNA.
