# Supplementary figures and images for: Mutations in the interleukin receptor IL11RA cause autosomal recessive Crouzon-like craniosynostosis
Source: Mol Genet Genomic Med. 2013 Aug 19;1(4):223–37. doi: 10.1002/mgg3.28 (PMC3865590; doi:10.1002/mgg3.28)

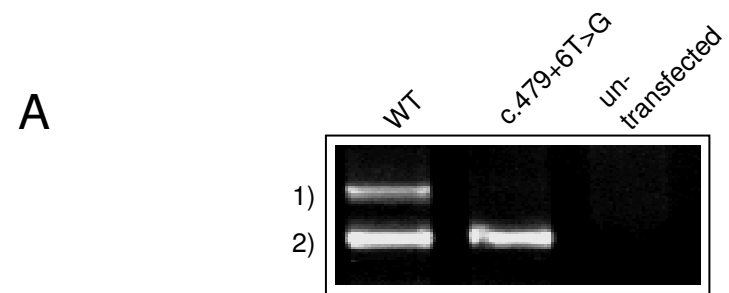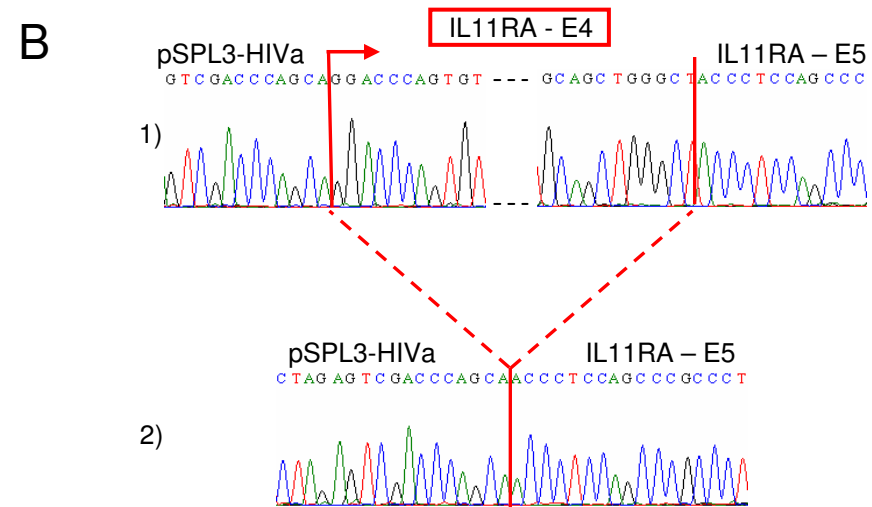

Supplement: Supplementary file 1 [file mgg30001-0223-SD1.pdf]
