# Supplementary material for: Mutations in the interleukin receptor IL11RA cause autosomal recessive Crouzon-like craniosynostosis
Source: Mol Genet Genomic Med. 2013 Aug 19;1(4):223–37. doi: 10.1002/mgg3.28 (PMC3865590; doi:10.1002/mgg3.28)
Supplement: Supplementary file 2 [file mgg30001-0223-SD2.doc]

**Supplementary Table 1:** Primer sequences used for amplification and sequencing of the *IL11RA* gene from genomic DNA

| primer | sequence | size (bp) | annealing temp. |
| --- | --- | --- | --- |
| IL11RA_E1A_F | GTG GGG GTG GAG GTG ATT G |  |  |
| IL11RA_E1A_R | TTA GCT GAC AGC TGC ACC AAG | 366 | 58°C (DMSO) |
| IL11RA_E1B_F | CTG GCC TTG GCT GTG TCT G |  |  |
| IL11RA_E1B_R | AGC ACG GAC CCT TCA GGA G | 407 | 58°C (DMSO) |
| IL11RA_2/3F | GAA AGG GGA CCT CAG GTC AG |  |  |
| IL11RA_2/3R | GGT GAG GAT TAC GGA CAG AGC | 721 | 58°C |
| IL11RA_4/7F | GCC AGT AAA AGG AAT TTG GG |  |  |
| IL11RA_4/7R | GCC TTC TCT GTG TGA AAT GG | 1095 | 58°C |
| IL11RA_8F | AGA TTT CCC CTC CCC TCT C |  |  |
| IL11RA_8R | ACC CAG CAC CCA GTA CAA AG | 434 | 58°C |
| IL11RA_9/11F | CAT GAG ACC TAG ACA GCT CCA C |  |  |
| IL11RA_9/11R | TGG GGA GGT AGT TCC AGA ATC | 1095 | 58°C |
| IL11RA_12/13F | ACC TCC CCA TAC CTC CAT CC |  |  |
| IL11RA_12/13R | GTC TCA CAG AAA TGG GCT CC | 915 | 58°C |
